# Effects of breathing maneuver and sitting posture on muscle activity in inspiratory accessory muscles in patients with chronic obstructive pulmonary disease

**DOI:** 10.1186/2049-6958-7-9

**Published:** 2012-06-20

**Authors:** Ki-song Kim, Min-kwang Byun, Won-hwee Lee, Heon-seock Cynn, Oh-yun Kwon, Chung-hwi Yi

**Affiliations:** 1 Department of Physical Therapy, The Graduate School, Yonsei University, 1 Yonseidae-gil, Wonju, Gangwon-do, 220-710, South Korea; 2 Division of Pulmonary Medicine, Department of Internal Medicine, Gangnam Severance Hospital, Yonsei University College of Medicine, Yonsei University Health System, 211 Eonju-ro, Gangnam-gu, Seoul 135-720, South Korea; 3 Department of Physical Therapy, Vision University, College of Jeonju, 235 Cheonjam-ro, Wansan-gu, Jeonju-si 560-760, South Korea; 4 Department of Physical Therapy, College of Health Science, Yonsei University, Institute of Health Science, Yonsei University, 1 Yonseidae-gil, Wonju, Gangwon-do, 220-710, South Korea

**Keywords:** Forward-leaning position, Inspiratory accessory muscles, Pursed-lips breathing, Quiet natural breathing, Sitting postures

## Abstract

**Background:**

To determine the influence of breathing maneuver and sitting posture on tidal volume (TV), respiratory rate (RR), and muscle activity of the inspiratory accessory muscles in patients with chronic obstructive pulmonary disease (COPD).

**Methods:**

Twelve men with COPD participated in the study. Inductive respiratory plethysmography and surface electromyography were used to simultaneously measure TV, RR, and muscle activity of the inspiratory accessory muscles [the scalenus (SM), sternocleidomastoid (SCM), and pectoralis major (PM) muscles] during quiet natural breathing (QB) and pursed-lips breathing (PLB) in three sitting postures: neutral position (NP), with armm support (WAS), and with arm and head support (WAHS).

**Results:**

Two-way repeated-measures analysis of variance was employed. In a comparison of breathing patterns, PLB significantly increased TV and decreased RR compared to QB. Muscle activity in the SM and SCM increased significantly in PLB compared to QB. In a comparison of sitting postures, the muscle activity of the SM, SCM, and PM increased in the forward-leaning position.

**Conclusions:**

The results suggest that in COPD, PLB induced a favorable breathing pattern (increased TV and reduced RR) compared to QB. Additionally, WAS and WAHS positions increased muscle activity of the inspiratory accessory muscles during inspiration versus NP. Differential involvement of accessory respiratory muscles can be readily studied in COPD patients, allowing monitoring of respiratory load during pulmonary rehabilitation.

## Background

Breathing training [1] and a sitting posture with a forward-leaning trunk [2] have been advocated as therapeutic interventions in patients with chronic obstructive pulmonary disease (COPD) to relieve dyspnea and improve pulmonary function. Previous studies suggested that pursed-lips breathing (PLB) increased tidal volume (TV) [3] and reduced respiratory rate (RR) [4] in patients with COPD. Additionally, PLB has been shown to lead to increased rib cage movement and accessory muscle recruitment during inspiration and expiration in patients with COPD [5].

Relief from dyspnea is often experienced in patients with COPD by assuming a forward-leaning position [2]. Sitting with a forward-leaning trunk and resting the forearms on the thighs is a modified position for relaxation in chest physical therapy [6-10]. A previous study indicated increased end-expiratory level and active expiration in sitting with a forward-leaning trunk compared to sitting leaning back [11]. In addition to the forward-leaning position, placing the head and neck in proper alignment can reduce airway obstruction, helping to increase pulmonary function [12].

However, the effects of a forward-leaning position on inspiratory muscle activity remain unclear. A previous study showed decreased activity of the scalene and sternocleidomastoid muscles in a forward-leaning position [2]. In contrast, another study indicated that a forward-leaning position with arm support allowed accessory muscles (i.e., the pectoralis minor and major) to contribute significantly to rib cage elevation, and arm and head support contributed to inspiration in the forward-leaning position [1]. Based on these differing results in previous studies, there is no consensus with respect to the muscle activity of the inspiratory accessory muscles in the forward-leaning position.

Thus, this study was performed to compare the TV, RR, and activity of respiratory accessory muscles during quiet natural breathing (QB) and PLB in three different sitting positions: a neutral position (NP), with arm support (WAS) in a forward-leaning position, and with arm and head support (WAHS) in a forward-leaning position in patients with COPD.

## Methods

### Subjects

Twelve male subjects (age = 68.2 ± 8.2 years; weight = 60.4 ± 6.9 kg; height = 1.7 ± 0.4 m; body mass index = 21.3 ± 2.0 kg/m^2^) diagnosed with COPD were recruited from the Division of Pulmonary Medicine, Department of Internal Medicine, Gangnam Severance Hospital, Yonsei University College of Medicine, Yonsei University Health System, Seoul, Korea. All subjects were classified as stage 2 or 3 COPD (forced expiratory volume in 1 s percent predicted (FEV_1_ %pred) (50.75 ± 9.27)) by the GOLD criteria [13]. The demographic characteristics and pulmonary functions of subjects are presented in Table 1.

**Table 1:** Demographic characteristics and pulmonary functions of the study population

Parameters		Mean ± SD
	Age (y)	68.2 ± 8.2
	Height (cm)	168.3 ± 4.3
	Weight (kg)	60.4 ± 6.9
	BMI (kg/m^2^)	21.3 ± 2.0
	FVC (L)	3.4 ± 0.6
	FEV_1_ (L)	1.4 ± 0.3
	FEV_1_/FVC (%)	40.1 ± 4.9
	FEV_1_ (%Predicted)	50.8 ± 9.3
	PEF (L/sec)	4.1 ± 1.1
	PEF (%Predicted)	54.4 ± 14.0
	MVV (L/min)	53.9 ± 13.3
	MVV (%Predicted)	46.8 ± 9.3

BMI, body mass index; FEV_1_, forced expiratory volume in 1 second; FVC, forced vital capacity; MVV, minute ventilation volume; PEF, peak expiratory flow.

All subjects provided written informed consent. This study was approved by the Yonsei University Wonju Campus Human Studies Committee.

### Measurement tools

Inductive respiratory plethysmography and surface electromyography were performed using an Embla N7000 (Embla Systems, Broomfield, CO) to enable the simultaneous acquisition and recording of respiratory parameters and surface electromyographic measurements.

### Data collection

#### 1) TV and RR

TV and RR were measured by inductive respiratory plethysmography (Embla N7000; Embla Systems). The system consisted of two bands (Teflon-coated inductance) that measured changes in the cross-sectional area of the rib cage and abdomen, allowing determination of the respiratory phase (inspiration and expiration).

#### 2) Muscle activity

For surface electromyography, three pairs of Ag/AgCl electrodes and a reference electrode were affixed on the patient’s inspiratory accessory muscles, the scalene muscle (SM), sternocleidomastoid muscle (SCM), and pectoralis major (PM). The EMG unit of the Embla N7000 (Embla Systems) was used to measure the muscle activity of the inspiratory accessory muscles. Data are expressed as percentages of the reference voluntary contraction (%RVC). Muscle activity was measured during the inspiration phase. The inspiration phase was determined from the nasal pressure curve, measured using a nasal cannula and respiratory analysis software for inductive respiratory plethysmography, and Origin 8, a program that calculates the root mean square (OriginLab, Northampton, MA).

### Procedures

**Figure 1:**
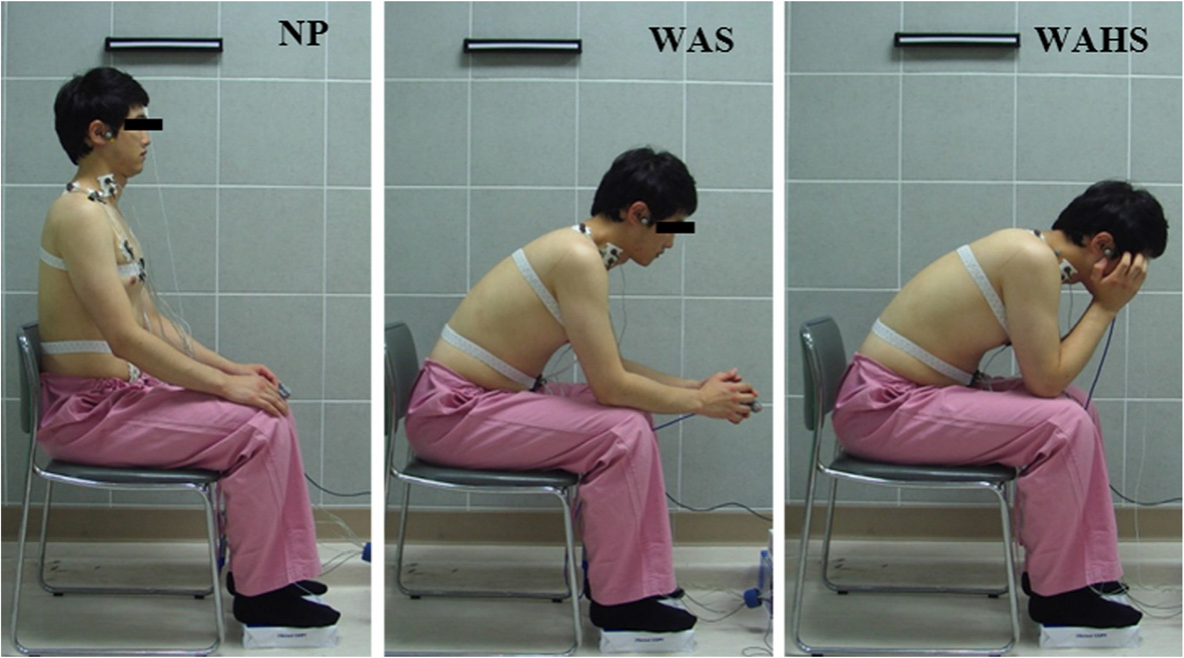
**Sitting postures.** NP, neutral position; WAHS, breathing training with arm and head support; WAS, breathing training with arm support.

Prior to enrollment in the study, all subjects received a training session for PLB. For the quiet natural breathing (QB) maneuver, patients were instructed to breathe in their normal habitual comfortable breathing style with no specific training. Breathing maneuvers and sitting postures to be tested (Figure 1) were selected by randomized ballots to eliminate any possible test order effect. All subjects felt relaxed and comfortable after a familiarization period of 5 min.

### Statistical analysis

The SPSS software (ver. 12.0; SPSS, Inc., Chicago, IL) was used for statistical analyses. Two-way (2 × 3) analysis of variance with repeated measures was employed to compare the two breathing maneuvers (QB, PLB) and three different positions (neutral, WAS, WAHS). In the case of significant differences between test positions, Bonferroni’s *post hoc* test was performed. In all analyses, p values < 0.05 were considered to indicate statistical significance.

## Results

### 1. TV and RR

The mean and standard deviation of tidal volume and respiratory rate for each respiratory maneuver and sitting posture are presented in Table 2. For TV, there was no interaction between breathing pattern and position (F = 0.132, p = 0.877). There was a significant main effect of breathing method (F = 90.017, p < 0.001), but there was no significant main effect of position (F = 0.837, p = 0.446). TV in PLB was significantly greater than that in QB. For RR, there was no interaction between breathing pattern and position (F = 1.462, p = 0.253). There was a significant main effect of breathing method (F = 50.702, p < 0.001), but there was no significant main effect of position (F = 1.387, p = 0.271).

**Table 2:** Tidal volume, respiratory rate and muscle activity for each respiratory maneuver and sitting posture (mean ± SD)

**Parameters**	QB			PLB		
	NP	WAS	WAHS	NP	WAS	WAHS
TV (ℓ)	0.7 ± 0.2	0.8 ± 0.2	0.8 ± 0.3	1.3 ± 0.1	1.3 ± 0.2	1.3 ± 0.2
RR (f/min)	18.1 ± 3.0	16.7 ± 3.4	16.5 ± 2.1	12.0 ± 0.6	12.0 ± 1.1	12.0 ± 0.9
SM (%RVC)	100.0 ± .0	104.4 ± 35.1	123.4 ± 25.2	118.4 ± 17.7	134.6 ± 31.2	150.1 ± 45.3
SCM (%RVC)	100.0 ± .0	113.1 ± 12.4	115.6 ± 13.7	115.8 ± 17.0	122.9 ± 24.0	131.0 ± 24.0
PM (%RVC)	100.0 ± .0	204.0 ± 89.0	161.2 ± 78.1	113.6 ± 20.5	271.1 ± 214.6	167.0 ± 69.2

NP, neutral position; PLB, pulsed lips breathing; PM, pectoralis major; %RVC, percentage of reference voluntary contraction; QB, quiet breathing; RR, respiratory rate; SCM, sternocleidomastoid muscle; SM, scalene muscle; TV, tidal volume; WAHS, breathing training with arm and head support; WAS, breathing training with arm support.

### 1. Muscle activity

**Figure 2:**
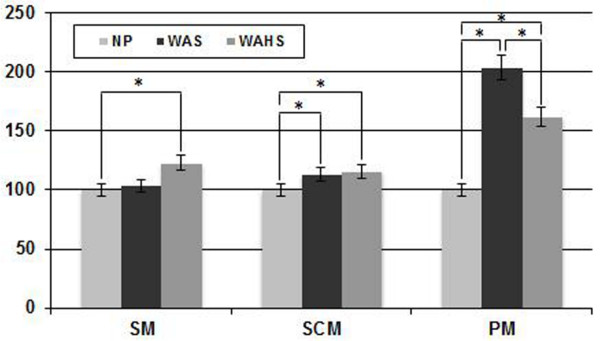
**Comparisons of %RVC among sitting postures.** NP, neutral position; PM, pectoralis major; %RVC, percentage of reference voluntary contraction; SM, scalene muscle; SCM, sternocleidomastoid muscle; WAHS, breathing training with arm and head support; WAS, breathing training with arm support (*p < 0.05).

The mean and standard deviations of %RVC for each respiratory maneuver and sitting posture are presented in Table 2. Comparisons of %RVC among sitting postures are presented in Figure 2. For scalene muscle (SM) activity, there was no interaction between breathing pattern and position (F = 0.830, p = 0.449). However, there were significant main effects of breathing method (F = 19.550, p = 0.001) and position (F = 7.466, p = 0.003). Muscle activity in PLB was significantly greater than that in QB. Bonferroni’s *post hoc* test showed that the muscle activity in WAHS was significantly increased compared to that in NP (p = 0.017). Muscle activity was not significantly different between WAHS and WAS (p = 0.070) or between WAS and NP (p = 0.495). For sternocleidomastoid muscle (SCM) activity, there was no interaction between breathing pattern and position (F = 0.650, p = 0.532), but significant main effects of breathing method (F = 5.751, p = 0.035) and position (F = 24.124, p < 0.001) were observed. Muscle activity in PLB was significantly greater than that in QB. Bonferroni’s *post hoc* test showed that the muscle activity in WAHS was increased significantly compared to that in NP (p = 0.001), and the muscle activity in WAS was increased significantly compared to that in NP (p < 0.001). No significant difference in muscle activity between WAHS and WAS (p = 0.154) was observed. For pectoralis major (PM) muscle activity, the results showed no interaction between breathing pattern and position (F = 1.138, p = 0.359) and no significant main effect of breathing method (F = 3.940, p = 0.073), but a significant main effect of position (F = 4.662, p = 0.037) was observed. Bonferroni’s *post hoc* test showed significant muscle activity differences among the three sitting positions used in this study. Muscle activity in WAS was increased significantly compared to that in NP (p = 0.025) and in WAHS (p = 0.029). Muscle activity in WAHS was increased significantly compared to that in NP (p = 0.039).

## Discussion

The findings of this study show that TV was significantly greater in PLB than in QB and that RR was significantly lower in PLB than in QB, supporting the research hypothesis. These results are consistent with previous studies indicating beneficial effects of PLB compared to QB for increasing TV [3] and decreasing RR [4,5].

The increased TV during PLB was probably due largely to the increased pressure in extra-thoracic airways and thus to a reduced cost of breathing due to decreased intrinsic positive end-expiratory pressure (PEEP). Many patients with COPD use PLB in an attempt to produce extrinsic PEEP to reduce lung hyperinflation and dyspnea [14]. The increased TV observed in our study may be explained by a recent study that reported that deflation of the abdomen and inflation of the rib cage contributed to increased tidal volume of the chest wall during PLB [15]. However, dyspnea scores in relation to postural changes and breathing maneuver were not measured in this study. A recent report showed that PLB slowed RR and increased TV both at rest and during exercise in COPD subjects; no subject complained of dyspnea at rest or during controlled exercise at 60 % of maximum exercise workload. However, dyspnea scores were increased with PLB in four of the eight COPD patients. [16]. PLB had no effect on minute ventilation, increased muscle activity of the abdominal muscles, and had a variable effect on dyspnea during volitional exercise in that study. Some studies have indicated that expiratory muscle recruitment is associated with a worsening of dyspnea [17,18]. No subject had received treatment with PLB or had used PLB voluntarily prior to this study. No patient complained of any increasing sense of dyspnea in each experimental trial because all subjects had a familiarization period with PLB and posture assumption before enrollment.

The diaphragm contains three types of muscular fibers (types I, II_A_, and II_B/X_), and the types of fiber vary in function with aging, type of exercise, and chronic respiratory load in patients with COPD [19]. A shift in diaphragmatic muscle fiber type toward slow-twitch, oxidative type I fibers, which are more fatigue-resistant, increases endurance, whereas protein degradation and a significant reduction in myosin content decreases muscular force-generating capacity [20]. Pulmonary hyperinflation shortens and flattens the diaphragm, altering the length of the diaphragm muscle fibers as well as their strength [21]. Concomitantly, the capacity of the diaphragmatic muscle to generate optimal pressure decreases due to the mechanical disadvantage in the length–tension relationship caused by hyperinflation [20].

Although there was no complaint of dyspnea during quiet breathing in this study, the effect of PLB in terms of relief of dyspnea could not be determined because we collected no data on expiratory muscle activity or dyspnea scales. Although favorable changes in TV and RR during PLB could not be shown to explain the positive effect in relief of dyspnea because of the limited data in our study, PLB is likely to reduce diaphragm activity and consequently may help to protect from fatigue of the diaphragmatic muscles during increased ventilatory conditions in patients with COPD [5,22,23].

In contrast to PLB, TV and RR did not differ significantly with sitting position. Thus, the research hypothesis regarding breathing position was not supported by the results of this study. These findings are consistent with a recent study by Bhatt et al. [6], who found no significant differences in FEV_1_, the ratio of forced expiratory volume to forced vital capacity (FEV_1_/FVC), maximum inspiratory pressure (MIP), maximal expiratory pressure (MEP), diaphragmatic movements during tidal breathing, or forced breathing in the sitting or supine positions, or sitting leaning forward with hands supported on the knees (tripod position) in patients with COPD. Kera and Maruyama [24] reported that TV did not change significantly according to sitting positions in 15 young adult men.

Posture can influence the degree of limitation on expiratory tidal flow according to changes in functional residual capacity (FRC) [25]. Recent studies described that slumped sitting decreases TV, FVC, FEV_1_, and peak expiratory flow compared to upright postures [26,27]. Another recent study reported that the effect of postural changes on respiratory movements of the chest wall had not been specifically addressed, and reported that single plane changes in sitting posture altered three-dimensional ribcage configuration and chest wall kinematics in seven healthy subjects during breathing while maintaining constant respiratory function [28].

Patients with severe COPD frequently lean forward, bracing their arms. A position bracing the elbows on a table increased ventilatory capacity significantly in four healthy men, and this effect could be helpful information for COPD patients, whose diaphragms are flattened and ineffective, as such patients depend more on the inspiratory muscles of the rib cage [29]. In contrast, bracing the arms impaired the function of the inspiratory muscles and reduced ribcage stability in six normal subjects, and these negative effects could not explain the improved capacity to sustain hyperpnea when the arms are braced [30].

Since the muscular force-generating capacity of the diaphragm is decreased in COPD and the effects of elbow bracing have differed among the past studies, in the present study we focused on changes in inspiratory accessory muscle activity related to changes in posture with or without elbow bracing to identify postural effects.

Although both the WAS and WAHS postures, characterized by the trunk leaning forward, might increase intra-abdominal pressure and decrease diaphragmatic excursion toward the abdominal cavity during inspiration, many patients with COPD adopt a sitting posture with the trunk leaning forward during conditions of an increasing sense of dyspnea [6,31], and athletic runners stand with the trunk forward, leaning, with hands on knees after finishing races to lessen ventilatory demand. Many researchers would like to identify sitting postures that may assist diaphragmatic function.

TV and RR did not differ significantly in relation to sitting position in this study. We had no data on inspiratory duty cycle or FRC according to different sitting posture; thus, our results do not provide complete information about postural changes in lung volumes.

The muscle activities of the SM and SCM in PLB were significantly greater than those in QB in this study. The fact that the SM and SCM attach between the cervical spine and the upper two ribs increased muscle activity in these muscles during inspiration in patients with COPD can be interpreted as an attempt to increase intrathoracic volume by elevating the upper ribs and sternum [31]. Increased SCM activity in PLB compared to QB is consistent with the results of a previous study indicating increased inspiratory rib cage expansion and recruitment of respiratory accessory muscles and reduced diaphragm recruitment during inspiration of PLB compared to tidal breathing [5].

In this study, muscle activities of the SM and SCM in a forward-leaning position, both WAHS and WAS, were greater than those in NP, even though the increased muscle activity of the SM in WAS relative to NP failed to reach statistical significance. Several mechanisms may explain these results. First, it is possible that increased activity of the SM and SCM overcame restricted downward movement of the diaphragm. The forward-leaning position results in increased intra-abdominal pressure by approximating the ribs to the pelvis, making it difficult for the diaphragm to descend caudally during inspiration [26,32]. A second possibility is the reversal of muscle action by the stabilizing force of the hand on the face in WAHS and of the forearm on the thigh in WAS. As the hands or forearms are stabilized, the sternum, clavicle, and rib cage can be pulled upward by the SM and SCM.

In this study, the PM showed the greatest muscle activity in WAS, followed by WAHS, and the lowest PM activity was observed in NP. These results may be explained by reversal of muscle contraction. When the distal limb segment is stabilized, the proximal limb segment can be mobilized. Decreased activity of the PM in WAHS, compared to that in WAS, may be attributable to increased SM and SCM activity in WAHS. However, muscle activities of the major muscles of respiration, including the diaphragm and intercostal muscles, were not measured, so the effects of changes in the activity of accessory muscles during different breathing maneuvers and of various positions on major muscle activity could not be determined in this study. Additionally, the effects of breathing maneuvers and sitting postures on pulmonary function in patients with COPD remain unclear because of limitations in the measurement of lung volume and dyspnea according to breathing maneuver and sitting posture. Further studies are required to measure the major muscles of respiration, including examination of various activities and conditions of ventilatory insufficiency, using various pulmonary parameters and dyspnea scales.

## Conclusions

In conclusion, these results suggest that in COPD patients PLB induced a favorable breathing pattern (as assessed by TV and RR) in comparison to QB. Additionally, WAS and WAHS induced increased activity of inspiratory accessory muscles during inspiration compared to NP in COPD. Differential involvement of the accessory respiratory muscles can be readily studied in COPD patients, allowing monitoring of respiratory load during pulmonary rehabilitation.

## Misc

Ki-song Kim and Min-kwang Byun contributed equally to this work.

## Competing interests

The authors declare that they have no competing interests.
